# Weather and risk of ST-elevation myocardial infarction revisited: Impact on young women

**DOI:** 10.1371/journal.pone.0195602

**Published:** 2018-04-09

**Authors:** Catherine Gebhard, Caroline E. Gebhard, Barbara E. Stähli, Foued Maafi, Marie-Jeanne Bertrand, Karin Wildi, Annik Fortier, Zurine Galvan Onandia, Aurel Toma, Zheng W. Zhang, David C. Smith, Vincent Spagnoli, Hung Q. Ly

**Affiliations:** 1 Montreal Heart Institute, Montreal, Canada; 2 Department of Nuclear Medicine, University Hospital Zurich, Zurich, Switzerland; 3 University Heart Centre, Freiburg-Bad Krozingen, Bad Krozingen, Germany; 4 Department of Anaesthesiology, University Hospital Basel, Basel, Switzerland; 5 Montreal Health Innovations Coordinating Center, Montréal, Canada; 6 Department of Medicine, Université de Montréal, Montreal, Canada; Osaka University Graduate School of Medicine, JAPAN

## Abstract

**Background:**

During the last decade, the incidence and mortality rates of ST-elevation myocardial infarction (STEMI) has been steadily increasing in young women but not in men. Environmental variables that contribute to cardiovascular events in women remain ill-defined.

**Methods and results:**

A total of 2199 consecutive patients presenting with acute ST-elevation myocardial infarction (STEMI, 25.8% women, mean age 62.6±12.4 years) were admitted at the Montreal Heart Institute between June 2010 and December 2014. Snow fall exceeding 2cm/day was identified as a positive predictor for STEMI admission rates in the overall population (RR 1.28, 95% CI 1.07–1.48, p = 0.005), with a significant effect being seen in men (RR 1.30, 95% CI 1.06–1.53, p = 0.01) but not in women (p = NS). An age-specific analysis revealed a significant increase in hospital admission rates for STEMI in younger women ≤55 years, (n = 104) during days with higher outside temperature (p = 0.004 vs men ≤55 years) and longer daylight hours (p = 0.0009 vs men ≤55 years). Accordingly, summer season, increased outside temperature and sunshine hours were identified as strong positive predictors for STEMI occurrence in women ≤55 years (RR 1.66, 95% CI 1.1–2.5, p = 0.012, RR 1.70, 95% CI 1.2–2.5, p = 0.007, and RR 1.67, 95% CI 1.2–2.5, p = 0.011, respectively), while an opposite trend was observed in men ≤55 years (RR for outside temperature 0.8, 95% CI 0.73–0.95, p = 0.01).

**Conclusion:**

The impact of environmental variables on STEMI is age- and sex-dependent. Higher temperature may play an important role in triggering such acute events in young women.

## Introduction

Cardiovascular disease is the leading cause of mortality in in the Western world. While cardiovascular mortality rates in men have steadily declined since the 1980s, the disease is becoming more common in women, with cardiovascular mortality rates in women currently exceeding those in men.[1–3] Most intriguingly, recent studies report a significant increase in hospitalizations for acute coronary syndromes (ACS) in women, with the most pronounced rise seen in young women aged 45–54 years admitted for ST-elevation myocardial infarction (STEMI).[2] Despite the excess cardiovascular risk in women, evidence to date has failed to adequately explore unique female determinants of cardiovascular disease. Indeed, women appear to possess differently weighted traditional cardiovascular risk factors than men for reasons that are currently unclear.[4] Further, sex-related cardiovascular risk factors such as polycystic ovary syndrome, premature menopause or a history of pre-eclampsia have all been shown to inflict a high risk for coronary artery disease (CAD) on women.[5] Most importantly, recent work indicates that non-traditional risk conditions such as socioeconomic and psychosocial factors may substantially contribute to the increasing risk noticed in younger women. In fact, depression and a greater perception of stress seem to impose an excess risk on young women as compared to men, thereby presenting a hidden biological risk in this population.[6, 7] The reasons for these unfavorable findings in women are unclear, however, a higher susceptibility to coronary microvascular dysfunction as well as an increased cardiac sympathetic activity in women have been suggested to account for their increased coronary risk.[6, 8, 9] Although fundamental biological differences in cardiac vulnerability to ambient stressors exist between men and women, there is currently a lack of sex-specific data on the impact of meteorological parameters on cardiovascular health, in particular in patients presenting with acute STEMI. In addition, while previous studies in Canada have focused on the effect of snowfall on cardiovascular hospitalisations [10, 11], data on the short-term interplay between environmental variables and the event of an acute coronary plaque rupture are lacking.

Thus, we analysed the association between environmental parameters and the incidence of acute STEMI in female and male patients admitted to a tertiary care centre in Montréal, Québec, a place that is undoubtedly well known for its cold and extreme temperatures ranging between -40°C in winter and +35°C during summer time.

## Methods

### Study population

In this single centre study, we retrospectively analyzed the number of daily hospital admissions for acute STEMI at the Montreal Heart Institute, Canada, during a 4.5 year period (06/2010-12/2014). Monthly and seasonal distribution of STEMI admission rates was analyzed. STEMI diagnosis was made according to the ESC/ACCF/AHA/WHF consensus document on the universal definition of non-fatal myocardial infarction.[12] Time of onset of chest pain and symptoms was obtained from patient self-reports recorded by emergency medical service or emergency department staff. Clinical data were retrospectively obtained by reviewing the full medical record of each patient and were fully anonymized before further analysis. The study complied with the declaration of Helsinki and was approved by the Research Ethics and New Technology Development Committee (CERDNT) of the Montreal Heart Institute. Due to the retrospective nature of the study, the need to obtain informed consent was waived by the ethics committee.

### Meteorological data

Data were obtained from the National Weather Service at Pierre-Elliott Trudeau International Airport (located 20km from downtown Montréal and covering the same geographic area) for the entire study period. Variables included in the analyses were as follows: daylight/darkness hours, 24-hour minimal, mean and maximal temperature (°C), day-to-day temperature variation, heating and cooling degree days (= measure of how much [°C], and for how long [days], the outside air temperature was below or above a certain level [day*temperature]), daily total rain (mm), daily total snow (cm), daily total precipitation (mm), daily snow on ground (cm), daily maximal humidity index (degree of discomfort the weather is causing to an average person by combining the effect of heat and humidity), daily maximal relative humidity (%), daily maximal wind chill (°C, perceived decrease in air temperature felt by the body on exposed skin due to the flow of air), and daily maximal wind speed (km/h). Humidity index, was classified according to level of discomfort caused (humidity index 20 to 29: little to no discomfort; 30 to 39: some discomfort; 40 to 45: great discomfort; >45: dangerous; heat stroke possible).

### Statistical analysis

Case data were analyzed by month, season and climate variable with χ2 test. Correlations between meteorological data and the frequency of STEMI were analyzed by the calculation of Spearman’s correlation coefficient. Poisson regression analysis assuming a log-link-function and a generalized linear model were used to assess the relative risks (RRs) and 95% confidence intervals (CIs) of incident STEMI associated with season and daily weather variables. The daily number of admissions for STEMI was set as dependent variable, while independent variables tested in this analysis were season, temperature>15°C, daylight>12hrs (all dichotomous variables) and maximal daily temperature (risk per single °C increase). In a multivariate analysis each of these variables was adjusted for total rain, total snow fall, relative humidity and wind speed. A multiple logistic regression model adjusted for cardiovascular risk factors (BMI, hypertension, diabetes, dyslipidemia, smoking and family history of CAD) was used to assess the effect of weather variables on clinical endpoints (periprocedural major adverse cardiovascular events [MACE], KILLIP class and TIMI flow on admission). Lag-correlations (lag1 = 24hrs) were performed to account for a potential time-lag phenomenon as previous studies have demonstrated delayed effects of environmental variables on cardiovascular events.[13] p values <0.05 were considered statistically significant. Statistical analyses were performed with IBM SPSS statistics version 23.0 (IBM, Armonk, NY, USA).

## Results

### Patient characteristics

A total of 2199 consecutive patients (25.8% women, mean age for women 67.5±13.4 years, mean age for men 60.9±11.5 years) were treated for a STEMI over the study period. Women admitted for STEMI were significantly older than their male counterparts (p<0.001). Ninety-seven percent of patients underwent primary percutaneous coronary intervention (PCI). In the male population, a higher proportion of smokers (52.0% vs 41.8%, p<0.001) was found, while female patients were more often hypertensive (57.9% vs. 45.9%, p<0.001). Among women, the highest proportion of smokers was found in patients <55 years old (72.7%, p<0.001). In addition, 50.5% of young women had a positive family history of CAD (p<0.001 vs other subgroups). Baseline patient and procedure-related characteristics stratified by gender and age (≤55 and >55 years) are shown in Table 1.

**Table 1:** Demographic and procedure-related patient characteristics stratified by sex and age (≤55 years; >55 years).

Demographic and procedure-related characteristics of the study population	All patients n = 2199	Women≤55 years n = 104	Women>55 years n = 465	Men≤55 years n = 511	Men>55 years n = 1119	p-value
Age (years), median[Q1;Q3]	61.7[54.0;71.0]	49.3[43.4;52.4]	70.7[63.9;80.7]	49.3[45.2;52.3]	64.4[59.4;72.4]	<0.001
BMI, kg/m^2^, median[Q1;Q3]	27.2[24.7;30.4]	27.4[23.5;31.4]	26.0[23.5;29.2]	27.6[25.1;30.8]	27.4[25.0;30.2]	<0.001
Hypertension, n (%)*	1013 (49.0)	34 (35.4)	274 (62.8)	146 (30.6)	559 (52.7)	<0.001
Diabetes, n (%)*	368 (17.8)	15 (15.6)	95 (21.9)	56 (11.7)	202 (19.2)	<0.001
Smoking, n (%)*	1022 (49.4)	72 (72.7)	149 (34.7)	333 (68.9)	468 (44.2)	<0.001
CAD family history, n (%)*	705 (34.7)	49 (50.5)	112 (26.7)	249 (52)	295 (28.5)	<0.001
Dyslipidemia, n (%)*	1254 (60.5)	54 (55.7)	263 (60.2)	263 (54.7)	674 (63.8)	<0.001
KILLIP class IV on arrival, n (%)	176 (8.0)	10 (9.6)	38 (8.2)	37 (7.2)	91 (8.1)	0.89
Cardiac arrest on arrival, n (%)	263 (12.5)	15 (15.2)	52 (11.7)	61 (12.6)	135 (12.6)	0.82
TIMI flow ≤1 at admission, n (%)	1545 (70.3)	64 (62.0)	328 (71.0)	385 (75.3)	777 (69.4)	0.08
TIMI flow ≤1 post PCI, n (%)	45 (2.1)	0 (0)	18 (3.9)	9 (1.8)	18 (1.6)	0.019

BMI, body mass index; CAD, coronary artery disease; PCI, percutaneous coronary intervention. Data are presented as median [interquartile **range, IQR] or n (%).**

* Data are missing in 127–170 patients.

### Seasonal and monthly distribution of STEMI admission

**Fig 1:**
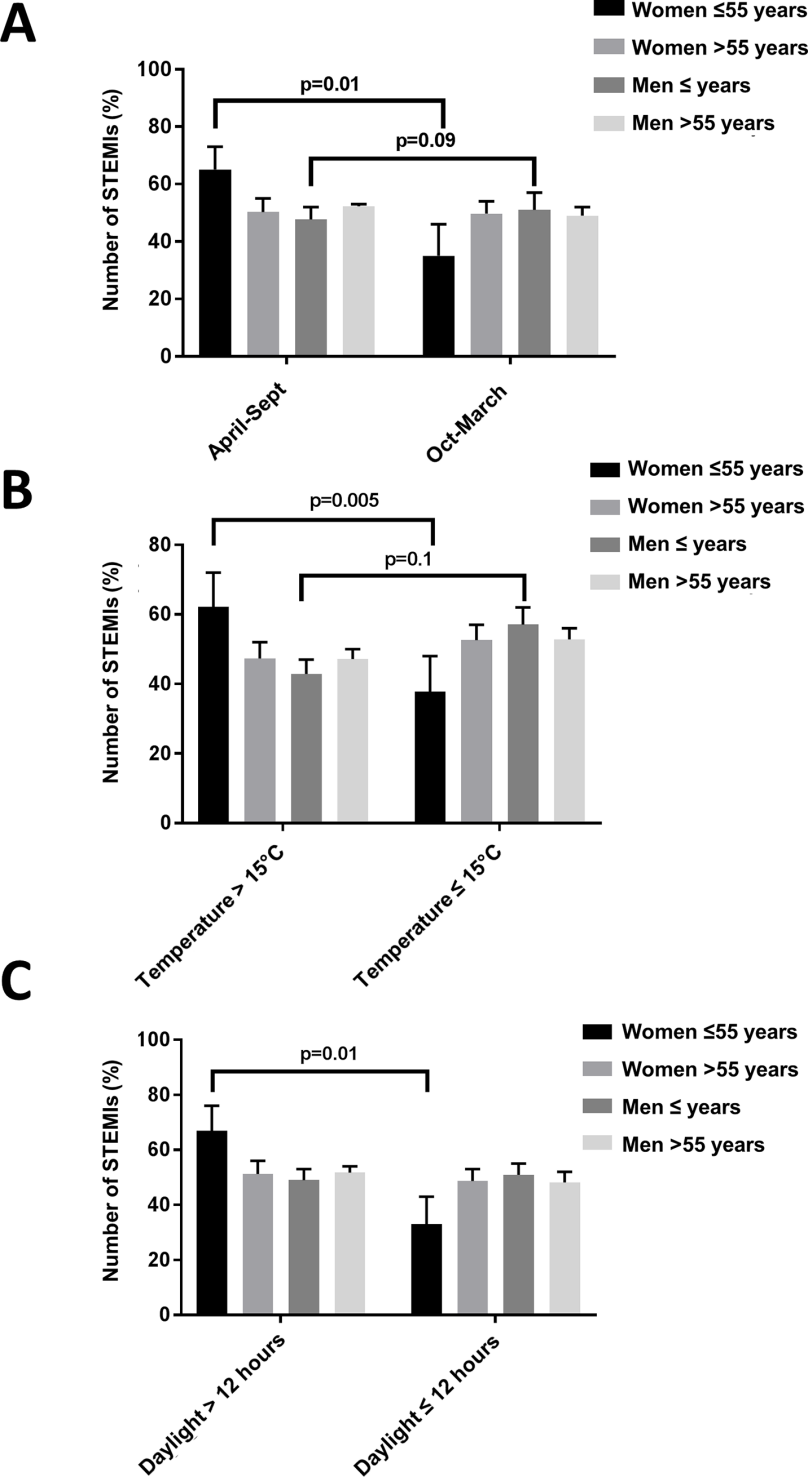
**A.** Seasonal distribution of ST-elevation myocardial (STEMI) infarction admission stratified by age and sex. **B.** Percentage of STEMI in age-and sex-specific subgroups stratified for outside temperature ≤15°C and >15°C. **C.** Percentage of STEMI in age-and sex-specific subgroups stratified for sunshine hours exceeding >12hrs/day. Data are presented as percentage (95% CI) within age/sex subgroup.

During the entire study period, there was no difference in access to the paramedic systems or ambulance service, as the Montréal Heart Institute serves as 24/7 primary PCI referral center for the North-East area of the City of Montréal as well as in suburban and rural areas in Northern Québec (longest distance for referral, 155 km). Prevalence of STEMI was higher in male patients irrespective of seasons (0.97 admissions/day in men and 0.34 admission/day in women, p = 0.002). A highly significant seasonal fluctuation in the incidence of STEMI in young women ≤55 years was observed, whereas this phenomenon was not seen in either men or older women >55 years (χ2 = 10.3, p = 0.02). While the number of STEMIs were equally distributed between April-September and October-March in older men (51.0% vs 49.0%, p = 0.57, Fig 1A) and older women (50.3% vs 49.7%, p = 0.55, Fig 1A), a marked increase in rates for STEMI during the summer was found in younger women ≤55 years (65.1% vs 34.9%, p = 0.01, Fig 1A). Conversely, a trend towards higher rates of STEMIs during winter months was observed in younger men ≤55years of age (47.7% vs 52.3%, p = 0.09, Fig 1A). Admission rates for STEMI in younger women peaked in August (17.5% of yearly admission), while the nadir occurred in February and March (3.9% of yearly admissions).

### Influence of individual meteorological parameters on STEMI admission rate

Over the study period, the median daily temperature was 9.5°C, ranging from −27.9°C to 35.6°C. In the overall study population, Poisson analysis identified snow fall > 2cm and maximal wind speed as significant predictors of STEMI rates (RR 1.02, 95% CI 1.00–1.04, p = 0.003 and RR 1.02, 95% CI 1.00–1.02, p = 0.019, respectively). When the population was stratified by sex, total daily snow fall > 2cm remained a significant predictor for STEMI occurrence only in men (RR 1.3, 95% CI 1.06–1.53, p = 0.01). The risk associated with snow fall was highest amongst elderly men >55 years (RR 1.4, 96% CI 1.1–1.7, p = 0.007). Further, a strong increase in STEMI admission in young women was observed when outside temperature exceeded 15°C, while this increase was not observed in older women and men (χ2 = 12.95, p = 0.005, Fig 1B). In young men, an opposite, nonsignificant trend was observed, with STEMI admissions increasing with lower temperatures (p = 0.11 vs older men, Fig 1B). Similarly, more STEMI admissions in young women were observed during days with longer sunshine hours (>12hrs), while this variation was not observed in other subgroups (χ2 = 11.14, p = 0.01, Fig 1C). Univariate and multivariate (adjusted for total rain, total snow fall, relative humidity and wind speed) Poisson analysis identified temperature, sunshine>12hrs, and summer season as significant positive predictors for STEMI in young women (Table 2); no such association was found in older men and older women. In younger men, a lower daily temperature was identified as a significant positive predictor of STEMI admissions (Table 2). Accordingly, heating degree days were associated with a significant risk reduction for STEMI occurrence in young women (multivariate Poisson regression: RR 0.97, 95% CI 0.95–0.99, p = 0.006), while cooling degree days showed a similar impact on young men (multivariate Poisson regression: RR 0.96, 95% CI 0.91–1.00, p = 0.049). Similar sex-specific trends and statistically significant relationships were found when a time delay of 24hrs (lag = 1) was added: maximal daily temperature >15°C: RR 2.11, 95% CI 1.2–3.3, p = 0.04 in young women and RR 0.81, 95% CI 0.52–0.95, p = 0.02 in young men (multivariate Poisson regression). In age- and sex-specific subgroups, no significant associations were found between STEMI admission rates and relative humidity, humidity index, diurnal and day-to-day temperature variations, wind speed, and precipitation (p = NS, data not shown).

**Table 2:** Poisson regression models depicting the effect of climatic variables on admission rates for ST-elevation myocardial infarction (STEMI) in women and men ≤55 years of age.

**Univariate Poisson Regression**	**Women≤55 years**			**Men≤55 years**			**All**		
**Climate variable**	**RR**	**95% CI**	**p-value**	**RR**	**95% CI**	**p-value**	**RR**	**95% CI**	**p-value**
**Spring/Summer-Season(April-Sept)**	1.66	1.12–2.46	0.012	0.86	0.72–1.02	0.09	0.96	0.88–1.04	0.32
**Maximal daily temperature >15°C**	1.70	1.15–2.51	0.007	0.80	0.67–0.95	0.011	0.95	0.87–1.03	0.21
**Daylight >12 hrs**	1.67	1.12–2.47	0.011	0.88	0.74–1.05	0.16	0.95	0.87–1.03	0.23
**Maximal daily temperature (per single degree [C°] increase)**	1.02	1.00–1.04	0.02	0.99	0.99–0.99	0.02	0.99	0.99–1.00	0.29
**Multivariate Poisson Regression**									
**Climate variable**	**RR**	**95% CI**	**p-value**	**RR**	**95% CI**	**p-value**		**95% CI**	**p-value**
**Spring/Summer-Season (April-Sept)**	2.40	1.06–5.43	0.036	0.85	0.59–1.24	0.403	1.03	0.86–1.23	0.77
**Maximal daily temperature >15°C**	2.00	1.1–3.7	0.029	0.75	0.57–0.98	0.034	0.97	0.85–1.10	0.62
**Daylight >12 hrs**	1.78	1.18–2.67	0.06	0.85	0.71–1.02	0.082	0.94	0.87–1.03	0.18
**Maximal daily temperature (per single degree [C°] increase)**	1.00	1.00–1.04	0.01	0.99	0.98–1.00	0.007	1.00	0.99–1.00	0.24

**Upper panel.** Univariate Poisson regression analysis testing the effect of season, temperature>15°C (categorical variable), daylight>12hrs (categorical variable) and maximal daily temperature (risk per single °C increase) on admission rates for STEMI. **Lower panel.** Multivariate Poisson regression analysis testing the effect of season, temperature>15°C (categorical variable), daylight>12hrs (categorical variable) and maximal daily temperature (risk per single °C increase) on admission rates for STEMI. Each variable was adjusted for total rain, total snow fall, relative humidity and wind speed. Due to collinearity daylight and temperature were not tested in the same model. RR, relative risk; CI, confidence interval.

### Impact of meteorological variables on clinical endpoints

**Fig 2:**
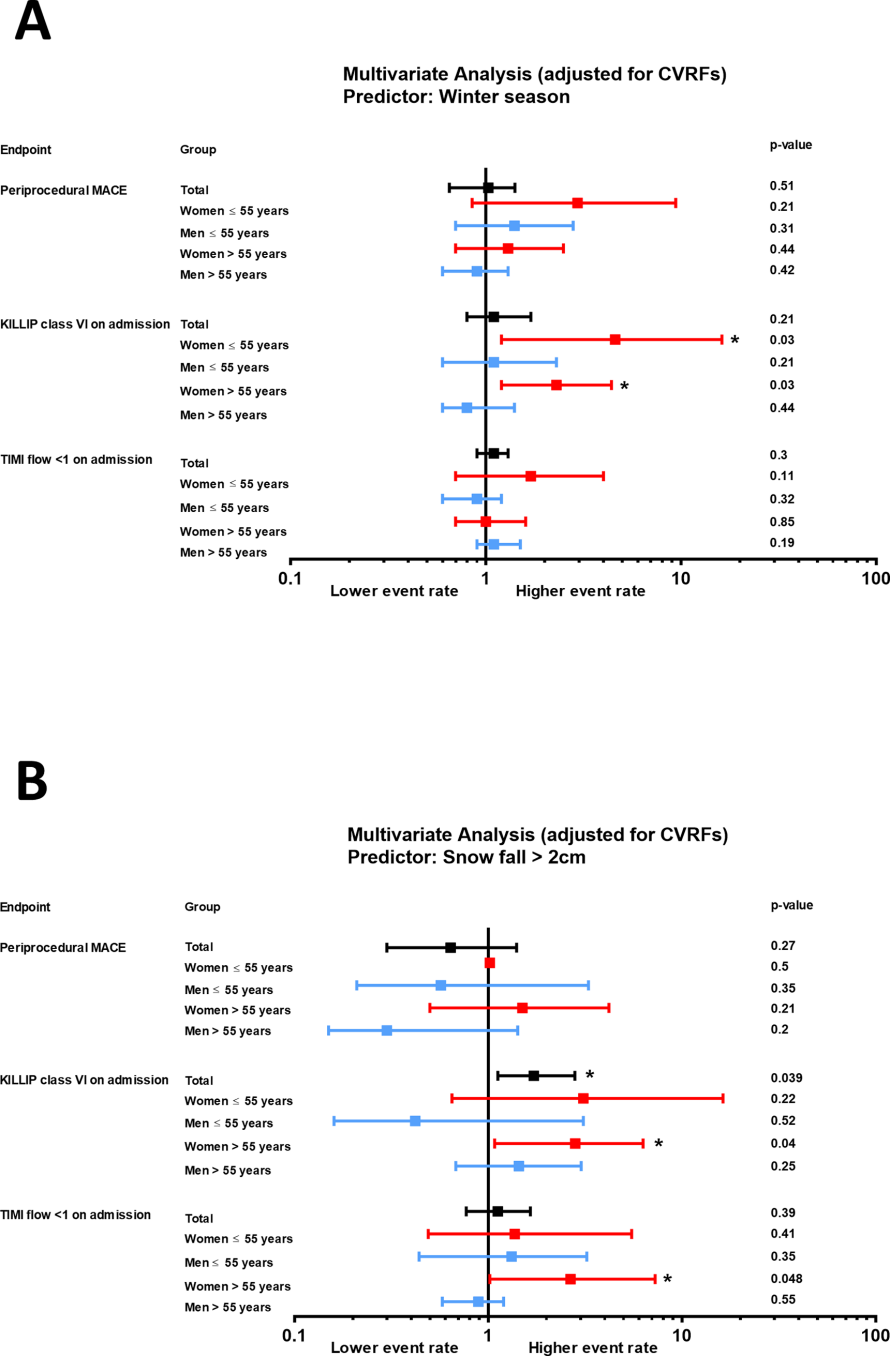
Relative risk and confidence intervals for the impact of **(A)** winter season and **(B)** snow fall > 2cm on clinical endpoints including periprocedural MACE (major adverse cardiovascular events), KILLIP class and TIMI flow at arrival. Results are provided for overall population (left) as well as for age- and sex-stratified subgroups. The multiple logistic regression analysis was adjusted for cardiovascular risk factors including body mass index, hypertension, diabetes mellitus, dyslipidemia, smoking and family history of coronary artery disease (CAD).

**Fig 3:**
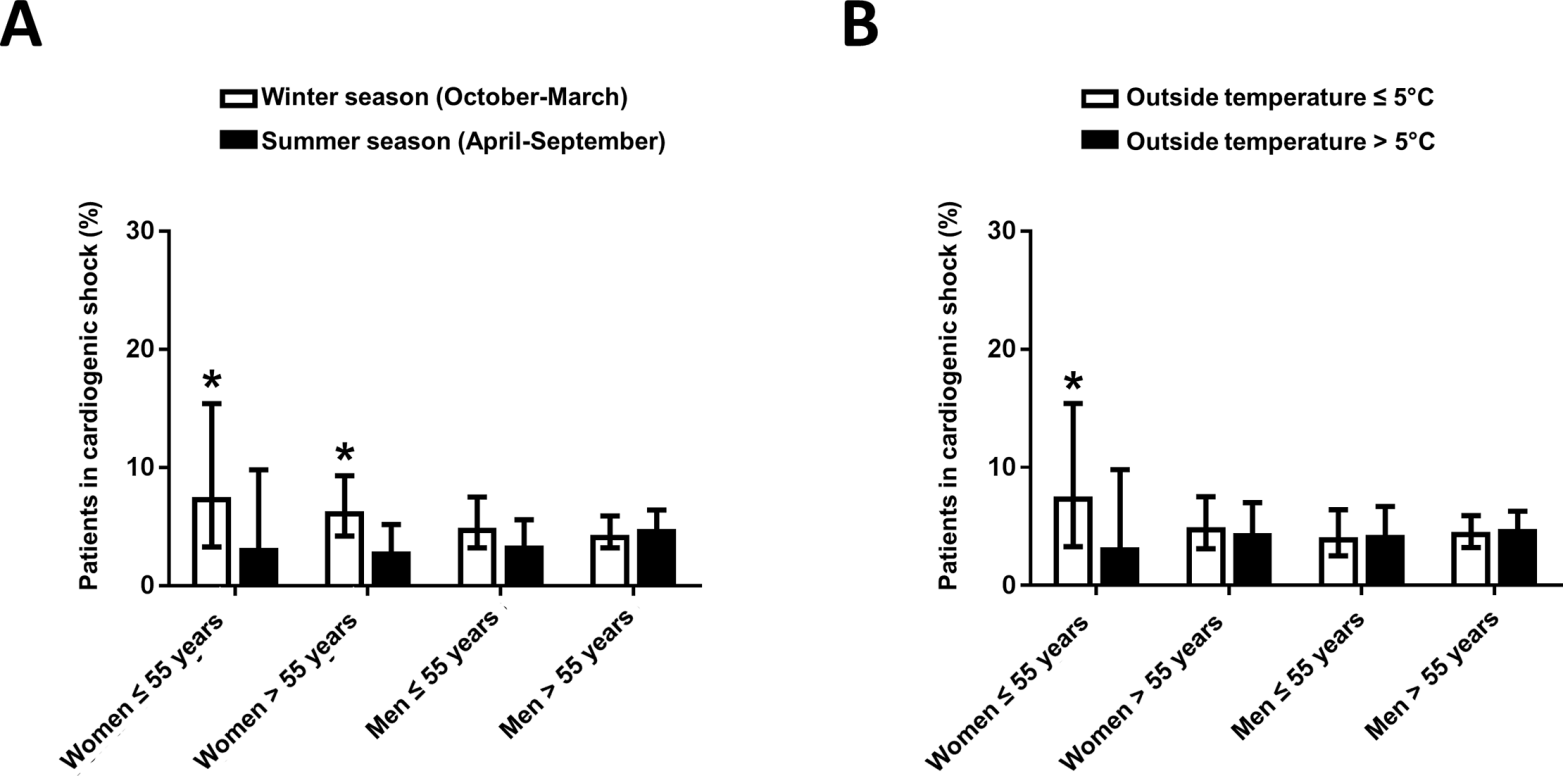
Effect of climatic variables on KILLIP class at arrival. **A.** Number of patients arriving at KILLIP class IV depending on season of the year. *p<0.05 vs. summer season. **B.** Mean outside temperature at KILLIP class IV vs. KILLIP class I-III arrival. Data are presented as percentage[95%CI]. *p<0.05 vs temperature>5°C.

Winter season was found to be a significant predictor of cardiogenic shock (KILLIP class IV) at arrival in women, but not in men (RR 2.46, 95% CI 1.38–4.70, p = 0.007 in women), with a pronounced effect documented in young women (RR 4.6, 95% CI 1.2–16.2, p = 0.03, Fig 2A). Accordingly, the proportion of women arriving in cardiogenic shock was significantly higher during winter as compared to summer (p<0.05 for younger and older women vs men, Fig 3A). Outside temperature was identified as a significant negative predictor of KILLIP class IV at arrival in younger women, but not in older women or men (RR 0.94, 95% CI 0.89–0.99, p = 0.039, data not shown). Accordingly, significantly more young women presented with KILLIP class IV STEMIs when outside temperatures were lower (Fig 3B). Snow fall>2cm significantly predicted the risk of KILLIP class IV on presentation, in the overall population (RR 1.72, 95% CI 1.12–2.80, p = 0.039, Fig 2B). In a sex-specific analysis, this association only remained significant in women (RR 2.90, 95% CI 1.20–6.78, p = 0.011), more so in elderly women (RR 2.81, 95% CI 1.08–6.30, p = 0.04, Fig 2B). Similarly, snow fall >2cm predicted TIMI flow≤1 at admission in women (RR 2.49, 95% CI 1.04–6.05, p = 0.044 in all women, RR 2.66, 95% CI 1.02–7.26, p = 0.048 in older women), but not in men (Fig 2B).

## Discussion

Our sex- and age-specific analysis is the first to identify an increase in outside temperature and sunshine hours as a significant and positive predictor for the occurrence of STEMI in young women but not in older women or men. Though increased STEMI rates in women were observed during the summer season, significantly more women arrived at the hospital in cardiogenic shock when outside temperatures decreased, an effect that was not observed in men.

Many previous studies have described winter peaks in cardiovascular hospitalisations, while the association between heat and cardiovascular risk is less consistent in the literature.[14] In our study, a significant association between decreasing temperatures, snow fall and increasing hospitalization rates for STEMI was observed in young men. This association, however, was weaker than the effect of higher temperatures on STEMI admissions seen in young women and, thus, did not translate into significant seasonal variations of STEMI admissions in men (p = 0.09). Possible explanations for these divergent findings might be variations in terms of geographical and climate areas assessed as well as in clinical endpoints and type of patients included. Indeed, while most previous studies report overall cardiovascular hospitalizations or mortality, inclusion criteria were narrowed to the definition of acute STEMI in the present study. This decision was based on the fact that the well documented onset of symptoms in this population allows assessing short term effects of environmental variables on coronary plaque rupture. Indeed, the importance of time and time-delays in the dose-response relationship of diurnal temperature and risk of cardiovascular events has recently been emphasized by a systematic review and meta-analysis where less pronounced effects of outside temperature on cardiovascular hospitalizations where observed when a one-day time lag was added to the analysis.[14]

Prior data report conflicting findings as to whether the risk associated with low or high temperatures applies equally to non-fatal and fatal events. In our study, we observed that women being hospitalized for STEMI during winter presented with higher rates of cardiogenic shock. Given that hospital admission rates for STEMI in women were higher during summer season, our observation confirms previous reports suggesting that cold stress might enhance the severity of an event rather than triggering its occurrence.[15] Accordingly, several studies suggest a stronger association between cold weather and mortality than with cardiovascular morbidity.[16–18]

It is currently unclear why young men, young women and older women showed opposing trends regarding the association between temperature and the incidence of STEMI in our study. Two earlier reports from Korea and Greece suggest a higher resistance of men to changes in air temperature and relative humidity as compared to women.[19, 20] Prior studies also indicate that younger people are more exposed to extreme temperatures than older subjects, because of work or other physical activities.[13] Indeed, although summer time is generally considered as a recreational time of the year, previous studies have reported increases in the rate of myocardial infarction and cardiovascular mortality associated with heat.[13, 21, 22] Thus, it is tempting to hypothesize that higher STEMI rates in young women during summer time might be the response to an increased exposure to thermal stress due to more outdoor time. In contrast, snow fall was identified as a significant and positive predictor of STEMI occurrence only in men indicating an enhanced exposure of the male population to the vigorous effect of snow removal. Of note, a higher susceptibility of women ≤50 years to ischemia and inflammation following a stress challenge as compared to men of similar age has recently been demonstrated and might put young women above a threshold of risk for abnormal responses to environmental influence.[6, 23–25]

Potential limitations of our study are as follows: first, only in-hospital patients were recorded, thus patients not surviving to reach our center (3–4%) were not accounted for in our analysis. Second, this is a retrospective study, with its inherent limitations, conducted in Québec; hence, the present findings may not be readily extrapolated to other populations or settings. Finally, our analysis is restricted to patients admitted to an interventional cardiology center, which may induce selection bias: STEMI cases managed solely medically were not included.

In our study carried out in a region with extreme weather conditions, an increased risk of STEMI was found in young women during the summer, but not in older women or men. This excess risk was largely attributable to high seasonal temperatures and longer sunlight hours. Given the steadily increasing rates of young women suffering from CAD, identifying specific triggers for the onset of STEMI in young women might be helpful to establish interventions specifically designed to address young women's stressors and could prompt further studies on the modulating effect of temperature on hormonal variations and its subsequent impact on the vulnerable plaque.
